# A Large Residual Vestibular Schwannoma Following Incomplete Resection: A Case Report With Literature Review

**DOI:** 10.7759/cureus.41314

**Published:** 2023-07-03

**Authors:** Saranya Ramsridhar, Chandini Rajkumar, Pooja Adtani, Khadijah Mohideen, Murali Balasubramaniam

**Affiliations:** 1 Oral Pathology, Sathyabama Dental College and Hospital, Chennai, IND; 2 Basic Medical and Dental Sciences, College of Dentistry, Gulf Medical University, Ajman, ARE

**Keywords:** tumor regrowth, incomplete resection, recurrent, vestibular schwannoma, acoustic neuroma

## Abstract

Vestibular schwannomas (VSs), also known as acoustic neuromas, are benign, slow-growing tumors. If not detected early or treated appropriately, these tumors can lead to complications such as pressure on adjacent intracranial structures that can affect vital functions. The present report discusses a rare case of a residual VS in a 46-year-old female patient. The patient was a known case of left-sided VS who underwent partial excision of the tumor four years ago and had complete hearing loss on the left side since then. She reported to the clinic with progressive headaches and imbalance while walking. Magnetic resonance imaging of the brain revealed a large left residual VS compressing the brainstem and cerebellum, which was completely excised, and the patient did well postoperatively. Incomplete resection of VS carries a significant risk of tumor regrowth, necessitating the importance of complete resection with periodic follow-ups.

## Introduction

Although vestibular schwannomas (VSs) are considered rare tumors, recent epidemiology reveals a lifetime prevalence of more than 1 case among 500 individuals [1]. VSs account for approximately 10% of primary intracranial tumors, 85% of cerebellopontine angle tumors, and 90% of intracranial schwannomas [2]. This neoplasm originates from Schwann cells ensheathing the axons of the eighth cranial nerve anywhere from the glial-Schwann junction up until the nerve terminations within the auditory and vestibular sense organs [3]. These tumors are usually unilateral or sporadic but rarely bilateral when associated with neurofibromatosis type 2 which occurs due to a defect on chromosome 22q12.2 at the location of the *neurofibromin 2* gene that encodes for merlin protein [4].

The most common clinical presentation of VS is unilateral hearing loss, followed by unilateral tinnitus, ataxia, vertigo, and headache. Larger tumors are associated with abnormal tandem gait, subjective facial weakness, abnormal facial sensation on examination, and headache [5]. Management of VS depends on the age of the patient, tumor size, and presenting symptoms. Management options include a wait-and-scan approach, radiation therapy, surgery, or a combination of all three. Radiation can be performed using stereotactic radiosurgery (SRS), stereotactic radiation therapy, and conventional fractionated radiation therapy. The most commonly used technique is SRS in which multiple beams are converged onto a delineated volume using cross-sectional imaging to minimize injury to adjacent tissues. Surgical approaches include retrosigmoid, middle cranial fossa, and trans-canal approaches, with each having its advantages and disadvantages [6].

This article focuses on the regrowth of a residual VS to a large size within a short duration after initial resection in a middle-aged female patient with associated brainstem and cerebellar compression which was treated by total surgical excision.

## Case presentation

**Figure 1:**
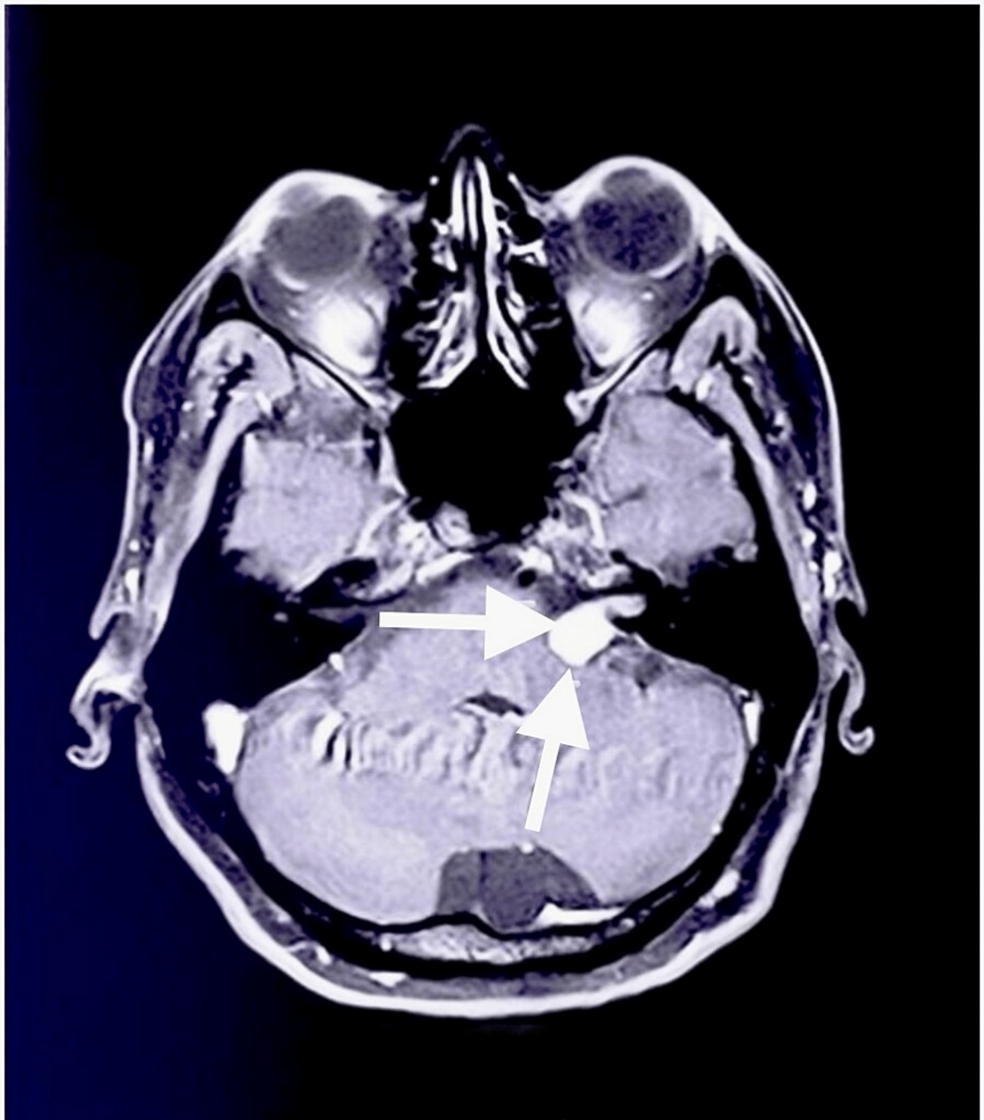
MRI of the brain showing the initial tumor (depicted by arrows). Intensely enhancing lesion in the left cerebellopontine angle with intracanalicular extension.

A 46-year-old female patient presented with a chief complaint of progressive headaches for the past eight months. She also complained of frequent vomiting, nasal regurgitation to fluids, change in voice, dizziness, and imbalance while walking in narrow passages and crowded areas for the past three months. She reported a history of left-sided VS for which she was operated on four years ago by left retrosigmoid suboccipital craniectomy and partial excision of the tumor was done with duraplasty using fascia. A small portion of the tumor remained on the seventh and eighth cranial nerves. The patient had complete left-sided hearing loss postoperatively. On physical examination, mild seventh, ninth, and tenth nerve paresis was present on the left side. Tandem walking was impaired. Left-sided horizontal gaze-evoked Bruns nystagmus was present. MRI of the brain (plain and contrast) of the initial tumor showed a 2.2 × 1.3 cm intensely enhancing lesion in the left cerebellopontine angle with intracanalicular extension (Figure 1).

**Figure 2:**
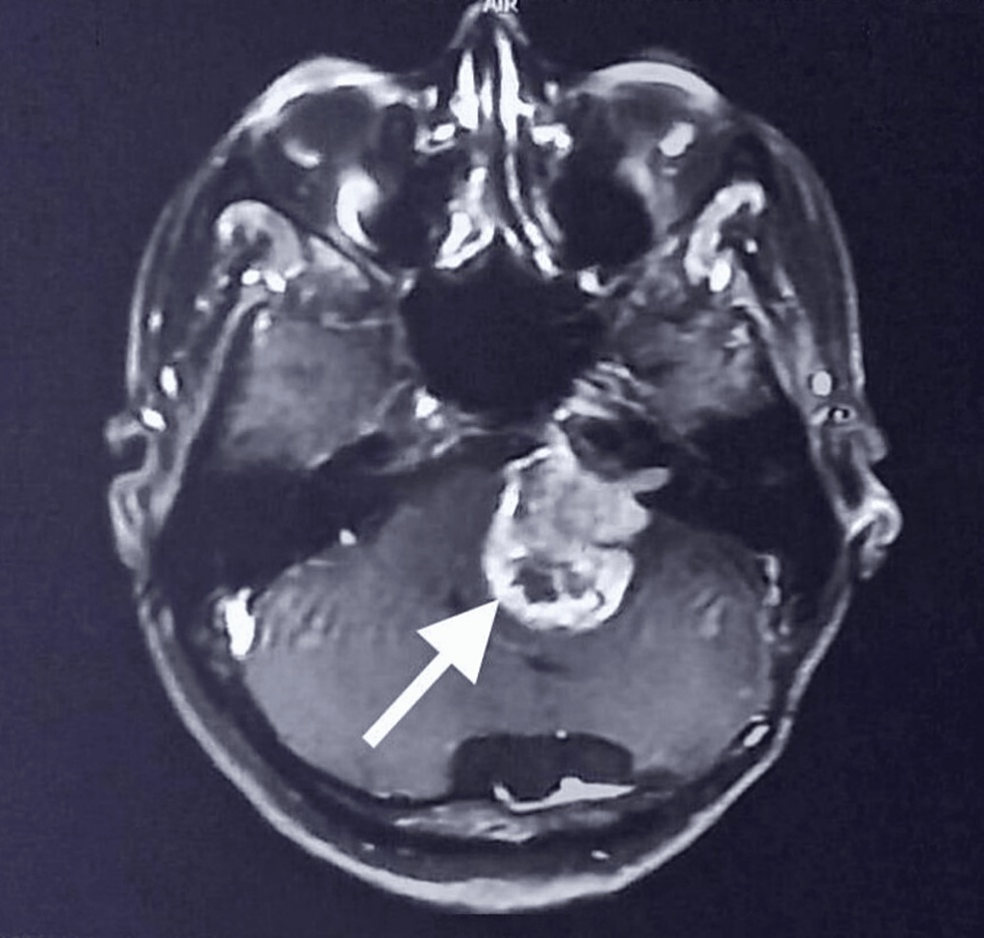
MRI of the brain showing regrowth of the residual tumor (depicted by arrow). A large heterogeneous enhancing lesion at the level of the internal auditory meatus.

MRI of the brain (plain and contrast) of the recurrent case revealed a 3.5 × 3.7 × 4 cm heterogeneously contrast-enhancing left cerebellopontine angle mass. There was no extension into the internal auditory meatus (Figure 2).

**Figure 3:**
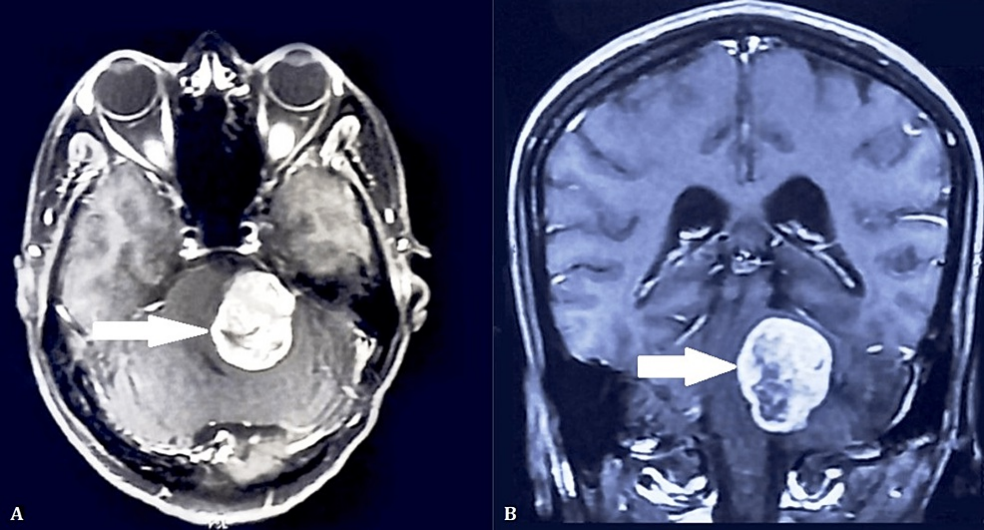
MRI of the brain showing regrowth of the residual tumor (depicted by arrow). A (axial view) and B (coronal view) revealing a large heterogeneous, enhancing lesion in the left cerebellopontine angle with compression of the brainstem and cerebellum.

The mass compressed the brainstem and the cerebellum on the left side (Figure 3). There was minimal hydrocephalus and a small degenerative postoperative cerebellar cyst at the superolateral aspect.

The patient was admitted, and the nature of the disease, the need for surgical re-exploration, and the pros and cons of the surgery were explained to the patient and her relatives. After obtaining informed consent, she underwent re-exploration of the left retrosigmoid suboccipital craniectomy and total excision of the large left residual VS under general anesthesia.

On gross examination, the tumor appeared well-circumscribed and encapsulated. The cut surface was yellowish-white in color. Areas of hemorrhage and cystic changes were seen, which were suggestive of degenerative changes. Histopathological sections were stained with hematoxylin and eosin and viewed at magnifications of 4×, 10×, and 40×.

**Figure 4:**
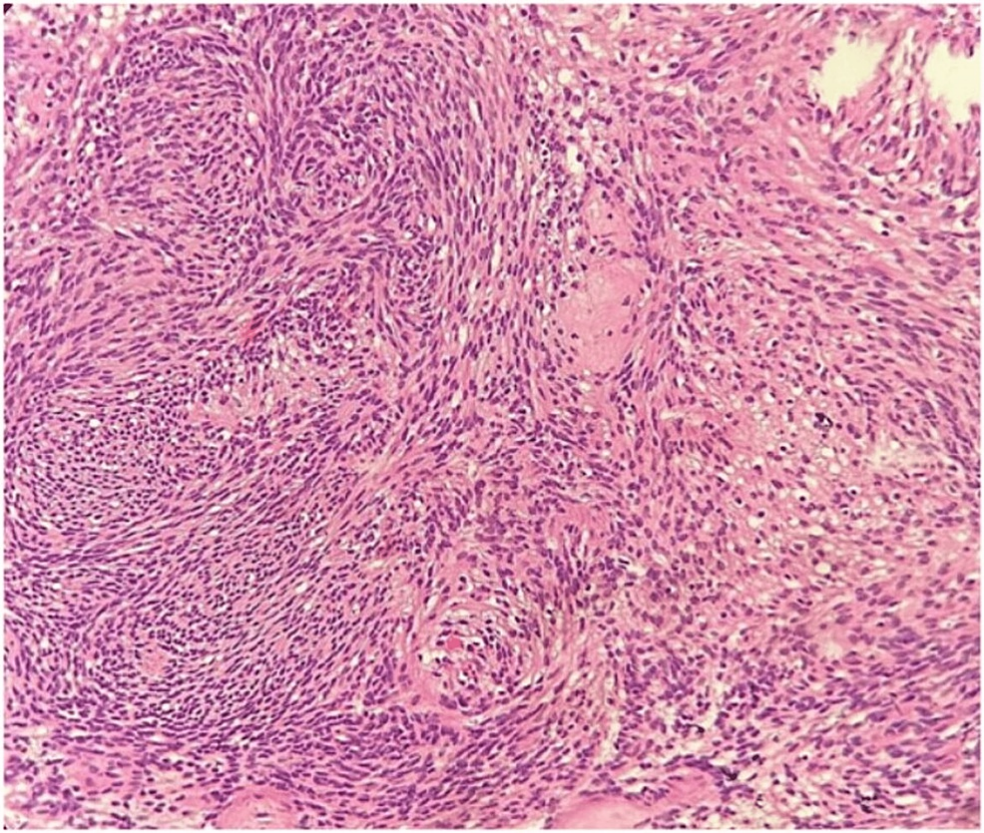
Histopathological section stained with hematoxylin and eosin at 40× magnification. Image showing spindle-shaped neural cells arranged in Antoni A pattern with Verocay bodies and less organized, hypocellular Antoni B pattern.

**Figure 5:**
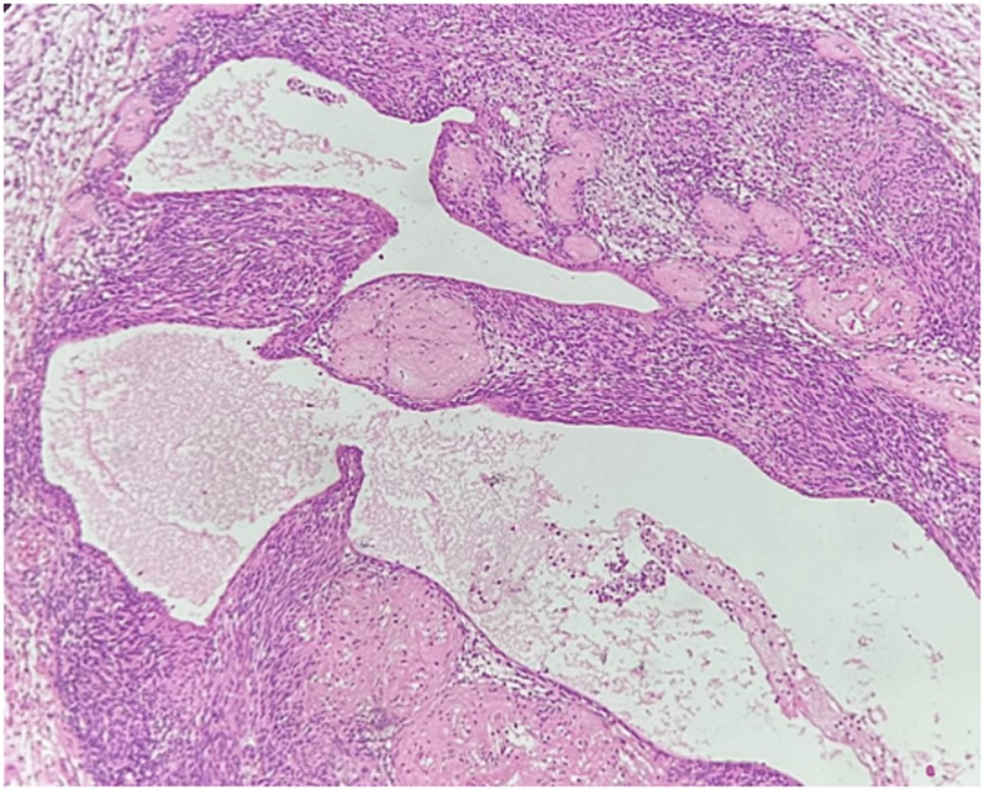
Histopathological section stained with hematoxylin and eosin at 10× magnification. Image showing hyalinization of blood vessels and microcysts.

**Figure 6:**
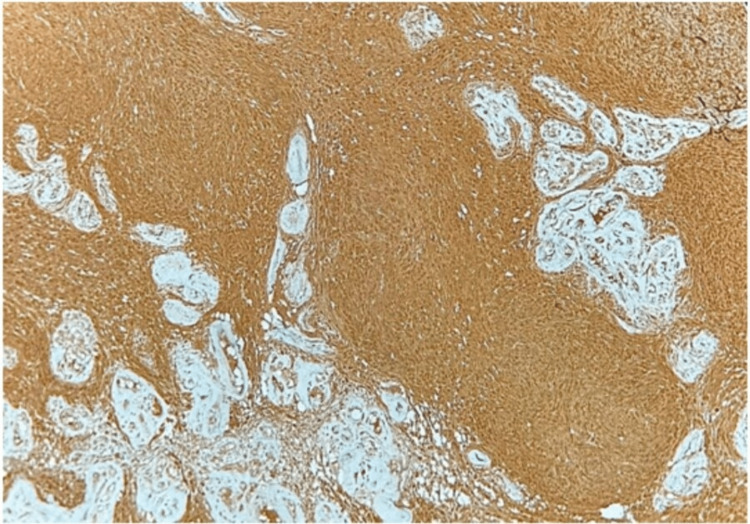
S-100 immunostaining at 10× magnification. Tumor cells showing strong reactivity for S-100 protein.

Microscopic features revealed a biphasic pattern. Cellular areas of streaming fascicles of spindle-shaped cells with elongated cytoplasmic processes and regular oval nuclei arranged in a palisaded or regimen pattern were observed exhibiting the characteristic Antoni type A. The spindle-shaped cells surrounded acellular, eosinophilic areas known as Verocay bodies. The adjacent microscopic tissue section demonstrated less organized, hypocellular areas with randomly distributed spindle cells interspersed within a loose, myxomatous connective tissue stroma with fibrillar collagen exhibiting the characteristic Antoni type B (Figure 4). In addition, thick hyalinized blood vessels and microcysts were observed, suggestive of degenerative changes (Figure 5). On immunohistochemical analysis of the tumor cells, a uniform intense nuclear and cytoplasmic staining for S-100 protein was observed (Figure 6).

Postoperatively, the patient experienced left seventh, ninth, and tenth cranial nerve paresis along with incomplete closure of the left eye. On discharge, the patient did well. Left ninth and tenth nerve paresis and left cerebellar signs completely improved, but the left lower motor neuron seventh nerve paresis was still present with incomplete closure of the left eye. No other deficits were observed, and the operative wound was seen to be healing well. The patient was asked to continue her medications regularly. She was advised to avoid any trauma to the operated site and avoid heavy lifting and straining. However, ongoing mobilization was encouraged to help recover the sense of balance and decrease dizziness. She was also advised facial physiotherapy and an occasional review with the ophthalmologist for the incomplete eye closure and was prescribed tear drops to avoid dryness. She was reviewed every month. Gradual improvement of left facial nerve paresis and left eye closure with every visit was observed, and there was no recurrence in the follow-up period of one year.

## Discussion

According to Dandy, “if any neurologic surgeon were asked to name the most difficult tumor to extirpate, his answer would doubtless be the acoustic tumor” [7]. However, over the decades, there has been substantial development in the management of VS with the advent of microsurgery and better diagnostic methods. Surgical resection of VSs has now become comparatively easy with minimal complications. Still, treating large lesions is considered complex, even in the hands of experienced surgeons.

The Koos grading scale (Table 1) is a frequently used classification system for VS that accounts for extrameatal tumor dimension and compression of the brainstem [8].

**Table 1:** Koos grading system for vestibular schwannomas.

Grade	Tumor extent
I	Intracanalicular
II	Minimal tumor extension into the cerebellopontine angle, <2 cm
III	Tumor occupies the cerebellopontine angle but does not displace the cerebellar trunk, <3 cm
IV	Large tumor with brainstem displacement, >3 cm

Radiosurgery is not advised for large tumors measuring >2.5 cm in diameter, brainstem dysfunction, significant mass effect, raised intracranial pressure, rapidly progressing symptomatology, and young patients (<50 years) with any tumor size. These lesions usually require surgical resection [9]. In this case, based on the tumor size, a retrosigmoid suboccipital craniectomy was performed both during the initial and recurrent phases. Compared to other microsurgical techniques, the retrosigmoid approach is considered safe and convenient for neurosurgeons, providing total cerebellopontine angle and brainstem exposure. This approach also offers low mortality and minimal morbidity with a high rate of functional facial nerve and hearing preservation, recovery of preoperative neurologic symptoms, and a low rate of complications with good functional outcomes [10-12].

The incidence of regrowth is high in cases of incomplete resection of VS requiring subsequent intervention, as in the present case. The risk factors for progression in VSs after incomplete resection include larger preoperative size, larger residual tumor volume, or irregular internal auditory canal expansion. Strict follow-up at shorter intervals is necessary in these patients to detect early progression [13].In their retrospective case review, Kashlan et al. reported 44% tumor regrowth among 39 patients who underwent incomplete resection of VS, thereby necessitating the importance of complete excision [14]. In a long-term follow-up study of 19 residual VSs, Kameyama et al. reported a regrowth in 10 VSs. Out of these 10 VSs showing regrowth, five with cyst formation showed rapid regrowth compared to the remaining five solid tumors which showed slow regrowth [15]. Thus, cyst formation is a major factor in the rapid regrowth of VS, as seen in the present case.

Our patient had unilateral hearing loss following the initial resection. Tumor size correlates with postoperative hearing preservation. Matthies et al. reported hearing preservation of 56% for T1 (intrameatal) tumors, 57% for T2 (extrameatal) tumors, 44% for T3 (medium-sized tumors), and 20% for T4 tumors with brainstem compression or dislocation [16]. Multiple factors may contribute to hearing loss in VSs, such as the growth pattern of the tumor, cochlear dysfunction, impairment of the auditory pathway and cortex, as well as genetic and molecular changes [17].

Facial nerve dysfunction was the main neurologic complication following the microsurgical removal of VS [2]. Translabyrinthine and retrosigmoid approaches with subtotal and near-total resections compared to gross total resection have shown better facial nerve outcomes postoperatively [18]. Surgery was the only treatment of choice in the present case based on the tumor size and associated complications. It was crucial to excise the tumor, leaving behind no fragments to prevent further regrowth. Hence, we must accept the compromise regarding facial nerve preservation keeping long-term tumor control in mind. However, surgical excision of acoustic neuromas can have physical and psychological consequences, thereby affecting the quality of life of patients. Hence, all patients should be well prepared before treatment, and adequate follow-up should be ensured after surgery.

## Conclusions

Although morbidity and mortality rates related to the management of VSs have improved significantly over the last few decades, radical surgical removal of large VSs continues to be challenging regarding facial nerve preservation and hearing loss. To avoid regrowth, VSs should be removed entirely whenever possible. If any tumor fragment is left behind for fear of damaging the facial or cochlear nerves, long-term surveillance is necessary with sequential clinical and MRI follow-up protocol. A well-experienced multidisciplinary team involved in the preoperative study of the imaging and clinical data, intraoperative neuromonitoring, and providing high standards of postoperative care are key factors for good prognostic outcomes in patients with large VSs.
